# Novel immune subtypes of lung adenocarcinoma identified through bioinformatic analysis

**DOI:** 10.1002/2211-5463.12934

**Published:** 2020-08-26

**Authors:** Fang‐lu Qin, Zhan‐yu Xu, Li‐qiang Yuan, Wen‐jie Chen, Jiang‐bo Wei, Yu Sun, Shi‐kang Li

**Affiliations:** ^1^ Department of Thoracic and Cardiovascular Surgery The First Affiliated Hospital of Guangxi Medical University Nanning China

**Keywords:** immune subtype, lung adenocarcinoma, prognosis, TCGA

## Abstract

The magnitude of the immune response is closely associated with clinical outcome in patients with cancer. However, finding potential therapeutic targets for lung cancer in the immune system remains challenging. Here, we constructed a vital immune‐prognosis genes (VIPGs) based cluster of lung adenocarcinoma (LUAD) from IMMPORT databases and The Cancer Genome Atlas. A transcription factor regulatory network for the VIPGs was also established. The tumor microenvironment of LUAD was analyzed using the ESTIMATE (Estimation of STromal and Immune cells in MAlignant Tumor tissues using Expression data) algorithm and single‐sample Gene Set Enrichment Analysis. The immune checkpoints and genomic alterations were explored in the different immune clusters. We identified 15 VIPGs for patients with LUAD and clustered the patients into low‐immunity and high‐immunity subtypes. The immune score, stromal score and ESTIMATE score were significantly higher in the high‐immunity subtype, whereas tumor purity was higher in the low‐immunity subtype. In addition, the immune checkpoints cytotoxic T lymphocyte associate protein‐4(CTLA4), programmed cell death protein‐1 and programmed death‐ligand were elevated in the low‐immunity subtype. The genomic results also showed that the tumor mutation burden was higher in the high‐immunity subtype. Finally, Gene Set Enrichment Analysis showed that several immune‐related gene sets, including interleukin‐2/STAT5 signaling, inflammatory response, interleukin‐6/Janus kinase(JAK)/signal transducer and activator of transcription 3 (STAT3) signaling, interferon‐gamma response and allograft rejection, were elevated in the high‐immunity subtype. Finally, high‐immunity patients exhibited greater overall and disease‐specific survival outcome compared with low‐immunity patients (log rank *P* = 0.013 and *P* = 0.0097). Altogether, here we have identified 15 immune‐prognosis genes and a potential immune subtype for patients with LUAD, which may provide new insights into the prognosis and treatment of LUAD.

## Graphical abstract

Here, we constructed a vital immune‐prognosis gene‐based cluster of lung adenocarcinoma (LUAD) from IMMPORT and The Cancer Genome Atlas databases. Immune checkpoints and genomic alterations were explored in the different immune clusters. Altogether, we identified 15 immune‐prognosis genes and a potential immune subtype for patients with LUAD, which may serve as the basis for future research into the prognosis and treatment of LUAD.

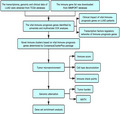

**Abbreviations**
AUC: area under the receiver operating characteristic curve
APC: Ag‐presenting cells
CCR: Cytokine and cytokine receptor
CDF: Cumulative Distribution Function
CTLA4: Cytotoxic T lymphocyte associate protein‐4
CI: confidence interval
DC: dendritic cells
ESTIMATE: Estimation of STromal and Immune cells in MAlignant Tumor tissues using Expression data
FDR: false discovery rate
GSEA: Gene Set Enrichment Analysis
HLA: human leucocyte antigen
IDC: immunogenic cell death
IFN: interferons
IL: interleukin
JAK/STAT3: Janus kinase/signal transducer and activator of transcription 3
LUAD: lung adenocarcinoma
MATH: mutant allele heterogeneity
NES: Normalized Enrichment Score
NOM: Nominal
NSCLC: non‐small cell lung cancer
P13K/AKT/mTOR: phosphatidylinositol‐3‐kinase (PI3K)/Akt and the mammalian target of rapamycin (mTOR)
PCA: Principal Component Analysis
pDC: plasmacytoid DC
PD‐1: programmed cell death protein‐1
PD‐L1: programmed death‐ligand
ROC: receiver operating characteristic
ssGSEA: single‐sample Gene Set Enrichment Analysis
TCGA: The Cancer Genome Atlas
TF: transcription factor
TMB: Tumor Mutation Burden
Tfh: T follicular helper
Th2: T helper type 2
TIL: tumor infiltrating lymphocytes
TME: tumor microenvironment
Treg: Regulatory T
VIPG: vital immune‐prognosis gene

Lung cancer, which includes non‐small cell lung cancer (NSCLC) and small cell lung cancer, is a disease with devastating consequences [1]. NSCLC includes lung adenocarcinoma (LUAD) and lung squamous carcinoma. LUAD is not only the most common histological subtype in lung cancer but also poses a higher risk for distant metastasis during all disease stages [2]. Despite diagnosis at an early stage and proper treatment with targeted therapy, chemotherapy and immune therapy, survival among patients with lung cancer is still limited [3]. Only a small proportion of patients with lung cancer is diagnosed early, and they have a 56% five‐year survival rate [4]. Although the targeted therapy and immunotherapy have greatly improved treatment for patients with lung cancer, identifying potential diagnostic markers, therapeutic targets in the immune system and immune cells that promote or inhibit the progression of lung cancer remains challenging [5]. Therefore, it is necessary to study new molecular markers of LUAD for potential therapeutic targets.

Recently, immunotherapies that activate the immune system to eliminate tumors [6] have shown broad prospects in treating NSCLC [7, 8]. For example, nivolumab, an antibody against programmed cell death protein‐1 (PD‐1), enhances the ability of cytotoxic T lymphocytes to kill NSCLC cells by blocking the interaction between advanced PD‐1 and its ligand, programmed death‐ligand (PD‐L1) [9]. The degree of immune cell infiltration between tumors and within tumors is different, and there are different barriers to neoantigen expression in different tumor microenvironments (TMEs) [10]. Immunotherapy stimulates the immune system to inhibit the growth and spread of tumor cells [11]. The influence of the TME on therapeutic response is becoming more and more important [12]. The TME consists of mesenchymal cells, immune cells, inflammatory mediators, endothelial cells and extracellular matrix molecules. Moreover, the TME also affects levels of gene expression in tumor tissue and clinical outcomes. In contrast, normal lung development depends on the activity of transcription factors (TFs) [13], and abnormal TF activity can lead to lung diseases. Therefore, it is particularly important to study the relationship between the TME and TFs, as well as prognostic markers of LUAD. Seo *et al*. [14] previously conducted the study focusing on the TME NSCLC based on the immune subtype. However, there were still many questions to be answered, for example, whether the intratumor heterogeneity and mutant allele tumor heterogeneity were affected by the different immunes subtype of LUAD. The potential role of the tumor mutation burden for the TME of the LUAD immune subtype should be uncovered. These discoveries of new immune subtype might help improve the treatment in patients with LUAD. Thus, a more comprehensive and systematic study is needed to get insight into the immune subtype of LUAD.

In this study, we identified 15 vital immune‐prognosis genes (VIPGs) and constructed novel immune subtypes for patients with LUAD from The Cancer Genome Atlas (TCGA) database. By combining clinical, genomic and transcriptomic data, we hope to uncover the immune mechanisms of LUAD and provide new evidence for the treatment of patients.

## Materials and methods

### Data standardization and preprocessing

Transcriptomic (fragments per kilobase of transcript per million mapped reads) and genomic data together with clinical data of LUAD were downloaded from TCGA database (https://cancergenome.nih.gov/) on April 1, 2020. Transcriptomic data included 594 samples in total (59 normal samples and 535 tumor samples). The limma r package was used to process the transcriptional data. Genes with averaged expression values close to zero were deleted. For the clinical analysis, samples with incomplete clinical traits and samples with a survival time <90 days were deleted, and a total of 456 LUAD samples were included for analysis.

### Exploration of VIPGs in LUAD

A list of 2498 immune genes were downloaded from the open web version of the IMMPORT database (https://IMMPORT.niaid.nih.gov). The differential immune genes were compared between normal samples and tumor samples by using Wilcoxon’s test [false discovery rate (FDR) < 0.05; logFC = 1, where FC is fold change]. Prognostic immune genes were first analyzed by univariate analysis (*P* < 0.05). Then VIPGs were identified by stepwise multivariable Cox proportional regression model, which acts as the independent prognostic factor for patients with LUAD.

### Clinical impact of VIPGs model in LUAD

The VIPGs were subsequently incorporated into prognostic models using their coefficients generated and expression data (${\text{Riskscore}}_{i}=\sum_{i}^{n}\beta_{i}\times \exp_{i}$). Each tumor sample was ranked according to the risk value from small to large to obtain the number of high‐risk and low‐risk patients. The expression levels of VIPGs involved in the construction of the model were displayed in the high‐ and low‐risk score groups [15]. Univariate and multivariate analyses, the Cox risk proportional regression model were used as predictors to determine which factors could be used as independent prognostic molecules [16]. The graphics were completed using the survival r package.

### TFs and immune gene‐regulatory networks

The comprehensive list of tumor‐related genes containing 318 TFs was obtained from the Cistrome Browser (http://cistrome.org/). TFs were intersected with the differentially expressed genes to identify the differentially expressed TFs. Correlation tests were conducted for VIPGs and TF (Pearson’s correlation: *R* ^2^ = 0.4, *P* < 0.05). cytoscape software (3.7.2) (http://www.cytoscape.org/) was used to visualize the regulatory relationship between prognostic immune genes and TFs.

### Immune clusters of LUAD based on the independent prognostic genes

After the determination of VIPGs of LUAD, we used the transcriptional expression data of VIPGs to explore the immune clusters of LUAD. The r package ConsensusClusterPlus was used to generate the consensus matrix plot by using the following parameters: 10 000 repeats and a maximum of six clusters. The 15 VIPGs were used for this clustering analysis.

### TME status of immune clusters

To evaluate the difference of TME in immune clusters, the enrichment fraction of each sample was calculated using single‐sample Gene Set Enrichment Analysis (ssGSEA) [17]. We downloaded 29 immune‐related gene sets containing immune cell types and immune‐related functions and pathways from the literature. By calculating the distance of each tumor sample, we compared immune cell types and immune‐related functions between low‐immunity and high‐immunity subtypes and visualized the differences using the pheatmap r package. Moreover, the ESTIMATE (Estimation of STromal and Immune cells in MAlignant Tumor tissues using Expression data) package was used to score the stromal and immune cells within malignant tumor tissues to predict the infiltration of nontumor lung cells. According to the gene expression data, the fundamental algorithm ESTIMATE is based on the ssGSEA. A stromal score was used to estimate the presence of stroma in tumor tissue, and the immune score was used to calculate the infiltration of immune cells in tumor tissue. As for the estimated score, it combines the stromal score and immune score to reflect the entire TME. Based on the immune clusters established by the VIPGs, we combined the different TMEs among the different immune statuses. In addition, we also obtained and compared the expression of PD‐1, PD‐L1 and CTLA4 from TCGA database.

### Alteration of somatic mutation in immune clusters of LUAD

The relevant somatic mutation data were obtained from TCGA database by using TCGA mutation package. To evaluate the intratumor heterogeneity of LUAD, we calculated mutant allele tumor heterogeneity of each patient by the maftools package. The tumor mutation burden was then calculated based on the TCGA_MC3 maf files. The landscape genomic alteration of LUAD was visualized by the oncoprint package.

### GSEA between immune clusters of LUAD

To investigate the alteration of the gene set, we performed the GSEA for the immune clusters. GSEA was analyzed by using the Java GSEA implementation. The gsea software and the hallmark gene sets were freely available (https://www.gsea‐msigdb.org/gsea/index.jsp). The number of permutations was set at 1000. The significance level of the gene sets was set at the absolute Normalized Enrichment Score (NES) > 1, Nominal (NOM) *P* < 0.05 and FDR *q* < 0.25.

### Statistical analysis

**Fig. 1:**
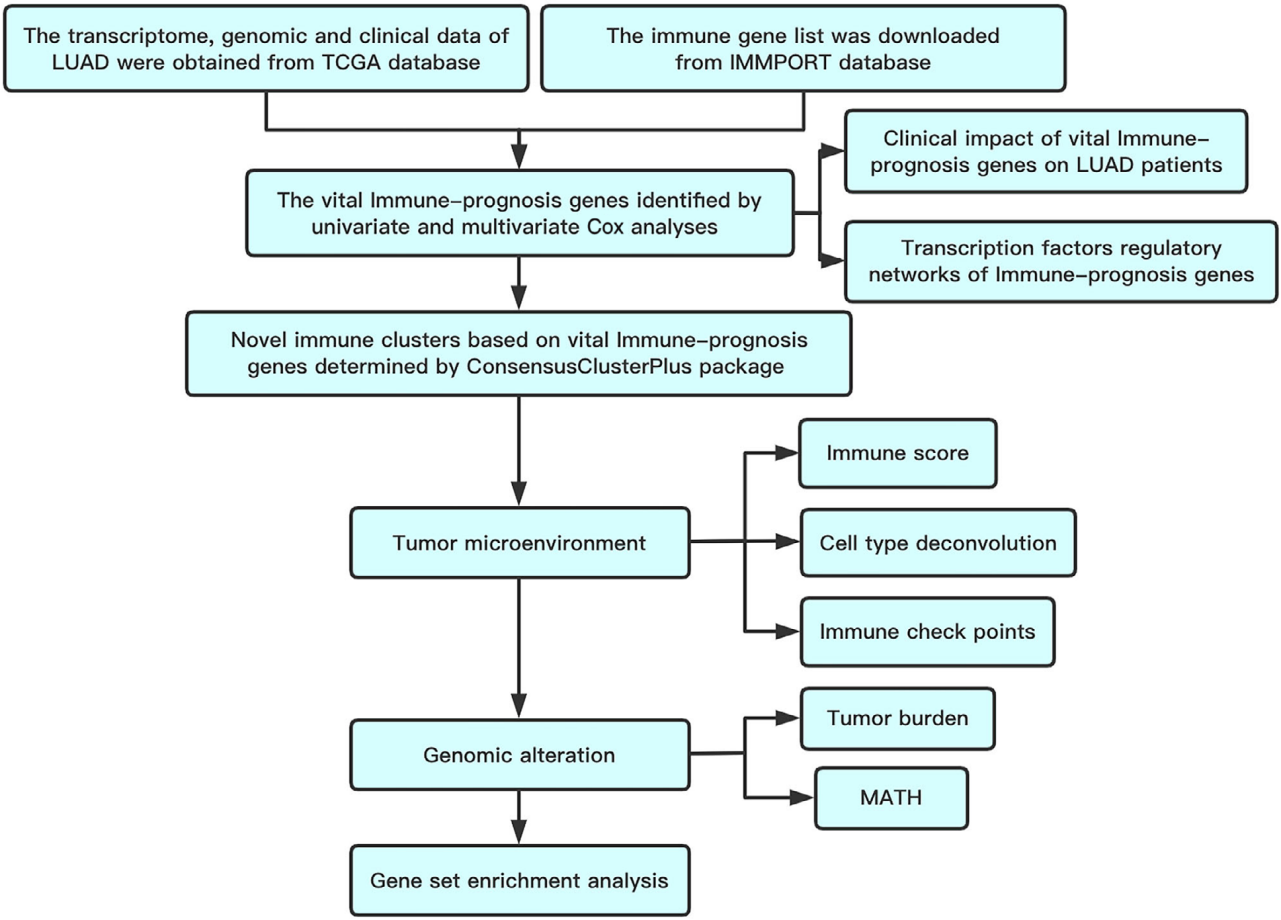
Workflow of the whole study design.

The Student’s *t*‐test was used to determine whether prognostic immune gene expression levels were correlated with clinical traits in the dichotomous categories. A χ^2^ test was used to test the statistical difference between categorical variable data. Log rank and Cox regression analysis were performed to determine the overall survival of patients and classify patients into high‐risk versus low‐risk groups based on median risk value. Factors that were analyzed included survival time, survival status and risk value. In addition, we calculated the sensitivity and specificity of our model using a receiver operating characteristic (ROC) curve (*P* < 0.05). The area under the ROC curve (AUC) was used to evaluate the prognostic effect of the model. A two‐sided *P* value <0.05 was set as the threshold of statistical significance. The analysis was conducted by using  r version 3.4.2 (2020‐04, https://www.r‐project.org/). The whole workflow of the study design is shown in Fig. 1.

## Results

### Determination of VIPGs in LUAD

A total of 594 LUAD samples (tumor versus normal samples) showed differential expression of genes. We identified 5112 up‐regulated and 1577 down‐regulated genes in these samples. Univariate analyses using the expression levels of immune genes identified 52 immune genes that were significantly related to prognosis, which facilitated the construction of prognostic immune gene models and regulatory networks. The 52 prognostic immune genes obtained by univariate analyses were used to establish a Cox proportional prognostic immune gene model. Finally, the list of prognostic genes was narrowed down to 15 immune genes, which were defined as VIPGs (Table 1).

**Table 1:** Independent prognostic value of VIPGs for patients with LUAD. Chr, chromosome.

Symbol	Chromosome location	Coefficient	HR (95% CI)
*S100A16*	Chr 1: 153606886–153613145	0.0014	1.0013 (1.0001–1.0027)
*CRABP1*	Chr 15: 78340324–78348230	0.0035	1.0034 (1.0007–1.0062)
*RBP2*	Chr 3: 139452884–139480747	0.0611	1.0629 (1.0283–1.0988)
*FGF2*	Chr 4: 122826708–122898236	0.3117	1.3657 (1.1645–1.6016)
*IGKV4‐1*	Chr 2: 88885397–88886153	‐0.0004	0.9996 (0.9993–0.9999)
*IGKV6D‐41*	Chr 2: 90069662–90070238	0.0231	1.0234 (1.0105–1.0364)
*SEMA4B*	Chr 15: 90160604–90229679	0.0047	1.0047 (1.0010–1.0099)
*FPR2*	Chr 15: 51752026–51770526	−0.3051	0.7371 (0.5718–0.9501)
*BDNF*	Chr 17: 27654893–27722058	0.225	1.2523 (1.0235–1.5323)
*IL11*	Chr 19: 55364389–55370463	0.1362	1.1459 (1.0346–1.2692)
*INHA*	Chr 2: 219569162–219575713	0.0074	1.0074 (1.0021–1.0128)
*ANGPTL4*	Chr 19: 8363289–8374373	0.0054	1.0054 (1.0008–1.0101)
*TNFRSF11A*	Chr 18: 62325287–62391292	0.2323	1.2615 (1.1391–1.3971)
*VIPR1*	Chr 3: 42489299–42537573	−0.135	0.8737 (0.7846–0.9730)
*SHC3*	Chr 9: 89005771–89178767	−0.2043	0.8152 (0.6694–0.9928)

### Clinical implication of VIPGs in patients with LUAD

**Fig. 2:**
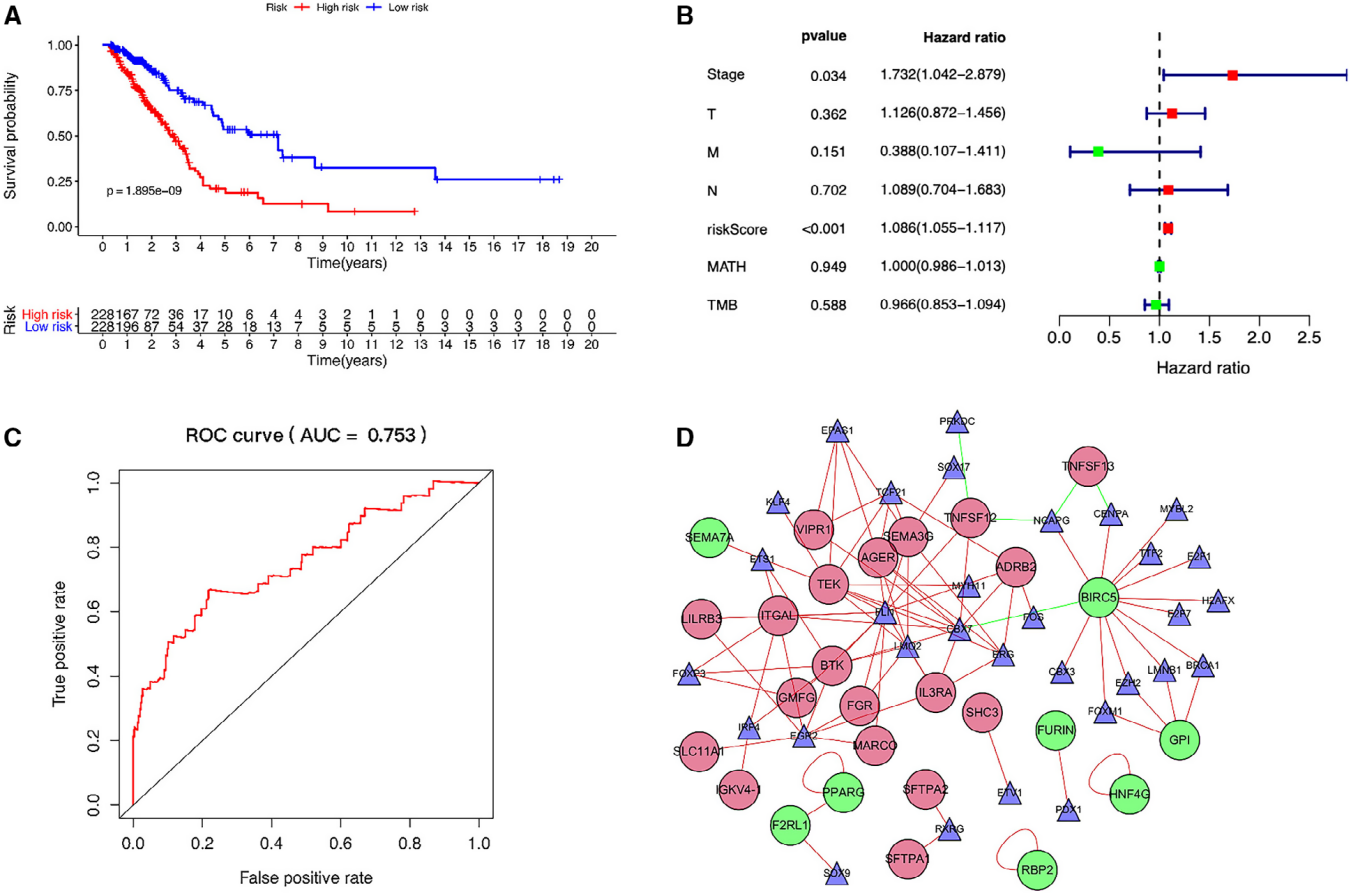
Clinical impact and TF regulatory networks of VIPGs for patients with LUAD. (A) Kaplan–Meier curves for the high‐ versus low‐risk groups. The survival rate of patients in the high‐risk group was significantly lower than the low‐risk group (*P* < 0.0001). (B) Forest plot showing the risk score of the VIPGs could serve as an independent prognostic factor for patients with LUAD. (C) The survival ROC revealed that the AUC of riskScore was 0.753, effectively predicting the 5‐year survival for patients with LUAD. (D) The TF regulatory networks of VIPGs. The circles represent immune genes, with red indicating high expression and green indicating low expression. The triangle represents TFs, which are linked by red lines for positive regulation and green lines for negative regulation. TMB, Tumor Mutation Burden.

To evaluate the entire clinical significance of VIPGs for patients with LUAD, we calculated the risk score of patients and divided patients into high‐risk and low‐risk groups. Survival time was measured using the number of years and the number of patients between the high‐ and low‐risk groups (*P* < 0.0001). Kaplan–Meier curves indicated lower survival rates among the high‐risk patients compared with the low‐risk patients (*P* < 0.05; Fig. 2A). The 5‐year survival rate of the high‐risk group was 18.81% [95% confidence interval (CI): 15.51–30.7%] and 53.4% for the low‐risk group (95% CI: 42.7–66.8%). Multivariate analyses showed riskScore [*P* < 0.001, hazard ratio(HR): 1.078, 95%CI:1.052–1.105], clinical stages (*P* = 0.018, HR: 1.829, 95%CI:1.108–3.019) were the significant independent prognostic factors for patients with LUAD (Fig. 2B). The survival ROC revealed that AUC of riskScore was 0.753, indicating a high level of reliability for the immune gene prognostic model for predicting the 5‐year survival of patients with LUAD (Fig. 2C). We also calculated the correlations between VIPGs and clinical characteristics using the prognostic model. Identifying differences in clinical traits of VIPGs will help determine the molecular mechanisms of tumorigenesis and development.

### Immune gene and TF regulatory network

All 70 TFs were differentially expressed; this finding intersected with genes that were differentially expressed genes between the tumor group and normal group. Of these 70 TFs, 41 were up‐regulated and 29 were down‐regulated. To find TFs that play a key role in regulating the immune response, we used gene‐regulatory network ontology to identify affected body immune processes and regulatory agencies [18]. Analysis of the regulatory network of immune genes and TFs identified 31 TFs related to prognostic immune genes. Only five TFs were associated with prognostic immune gene models. The regulatory networks of molecular interactions may play a critical role in the transcriptional control of VIPGs (Fig. 2D).

### Construction of the immune subtypes

**Fig. 3:**
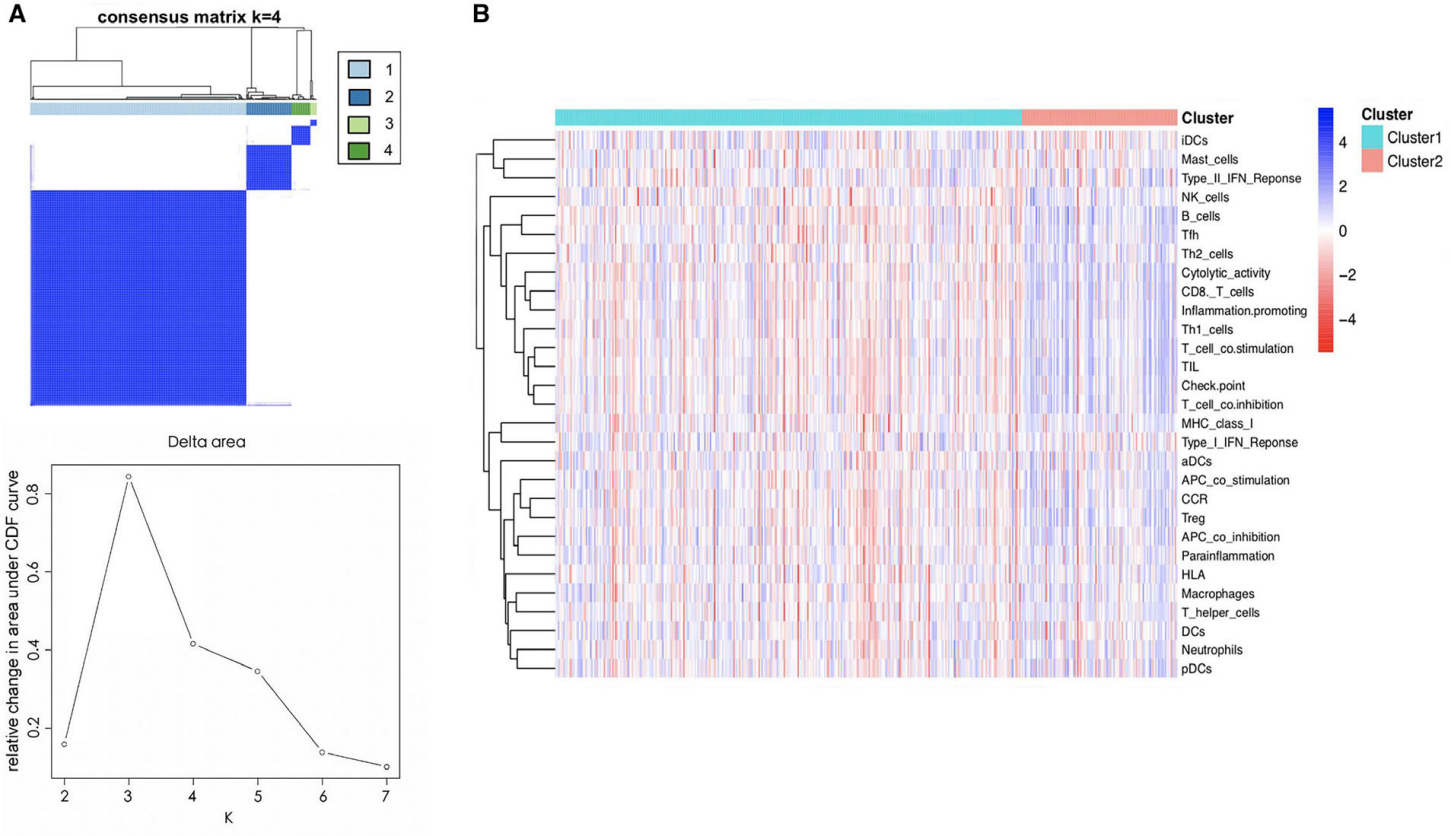
Immune subtypes based on VIPGs and their relationship with the TME. (A) Immune clusters based on VIPGs for patients with LUAD. A total of six clusters were generated by ConsensusClusterPlus, and the four‐cluster model was used for further analysis. The consensus matrix is sorted by consensus clusters, which are represented by the tree at the top of the heatmap. The colored rectangle between the trees marked cluster membership. (B) Heatmaps showed the immune activity of different cell types in the low‐ immune(cluster 1) and high‐ immune (cluster 2) groups.

Based on the clinical implication of VIPGs in LUAD, we then used the 15 VIPGs to cluster the patients with LUAD into different subtypes. As Fig. 3 shows, a total of four clusters were identified by the ConsensusClusterPlus. Then we divided the patients into the low‐immunity (cluster 1: a total of 356 patients with LUAD) and high‐immunity subtypes (clusters 2–4: a total of 100 patients with LUAD) based on the immune score derived from the ESTIMATE algorithm (Fig. 3A).

### TME in different immune clusters

**Fig. 4:**
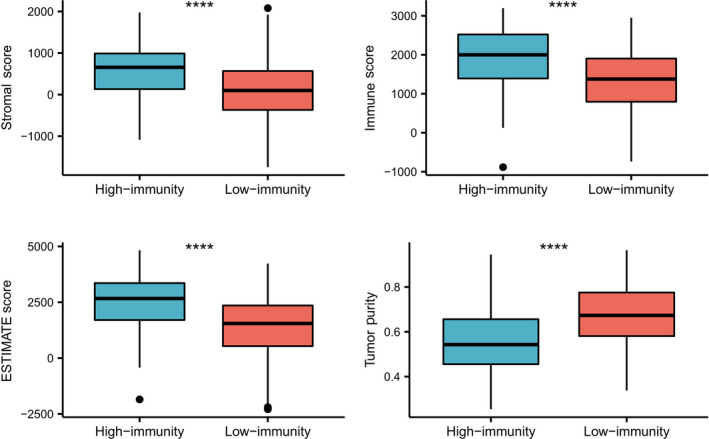
Distribution of tumor purity, ESTIMATE score, immune score and stromal score in high‐ versus low‐immunity groups. The estimated score, stromal score, and immune score were higher among the high‐immune patients compared with low‐immune patients. However, the purity of the tumors in the high‐immune group was lower compared with low‐immune patients. Furthermore, the estimated score of the high‐immune group was higher than that of the low‐immune group and was indirectly proportional to the tumor purity. The Student’s *t*‐test was used to compare the difference between high‐ and low‐immunity groups (*** *P* < 0.001).

**Fig. 5:**
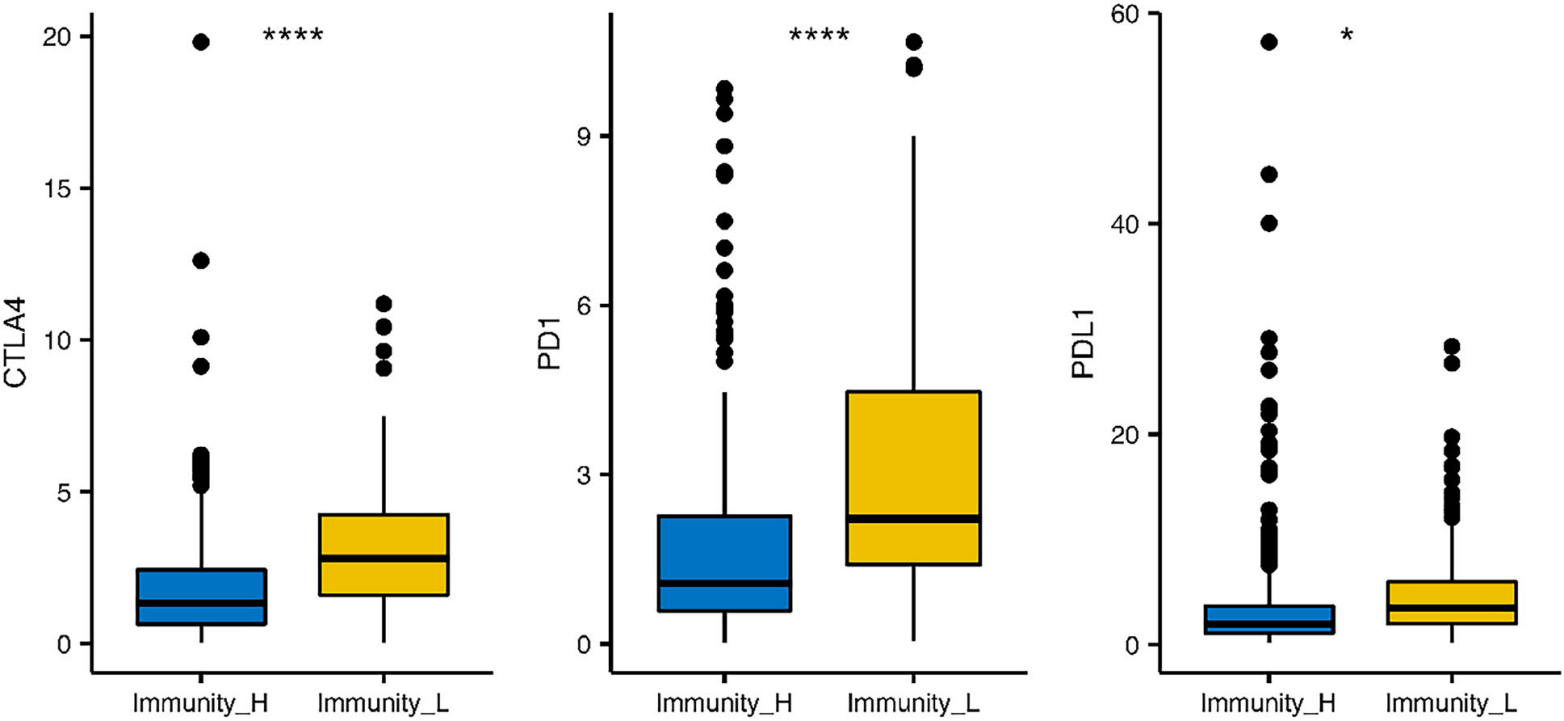
Boxplot of expression levels for the immune checkpoints in high‐ versus low‐immunity groups. The immune checkpoints CTLA4, PD‐1 and PD‐L1 were up‐regulated in the low‐immunity subtype, indicating the immunosuppression in the low‐immunity group. The Student’s *t*‐test was used to compare the difference between high‐immunity and low‐immunity groups (*P < 0.05; *** *P* < 0.001).

Immune cell infiltration was assessed by ssGSEA analysis using 29 immune gene sets with cluster 1 as the low‐immune group and clusters 2–4 as the high‐immune group. Among different immune cell types, the immune activity of the high‐immune group was higher than that of the low‐immune group (Fig. 3B). The ESTIMATE algorithm was used to calculate the immune score, stromal score and estimate score for the tumor tissues of the expression profile [19]. We concluded that the estimated score, stromal score and immune score were higher among the low‐immune patients compared with the high‐immune patients. However, the purity of the tumors in the high‐immune group was lower compared with low‐immune patients. Furthermore, the estimated score of the high‐immune group was higher than that of the low‐immune group and was indirectly proportional to the tumor purity (Fig. 4). In addition, the immune checkpoints CTLA4, PD‐1 and PD‐L1 were up‐regulated in the low‐immunity subtype, indicating the immunosuppression in the low‐immunity group (Fig. 5).

### Genomic alteration of the patient in immune clusters

**Fig. 6:**
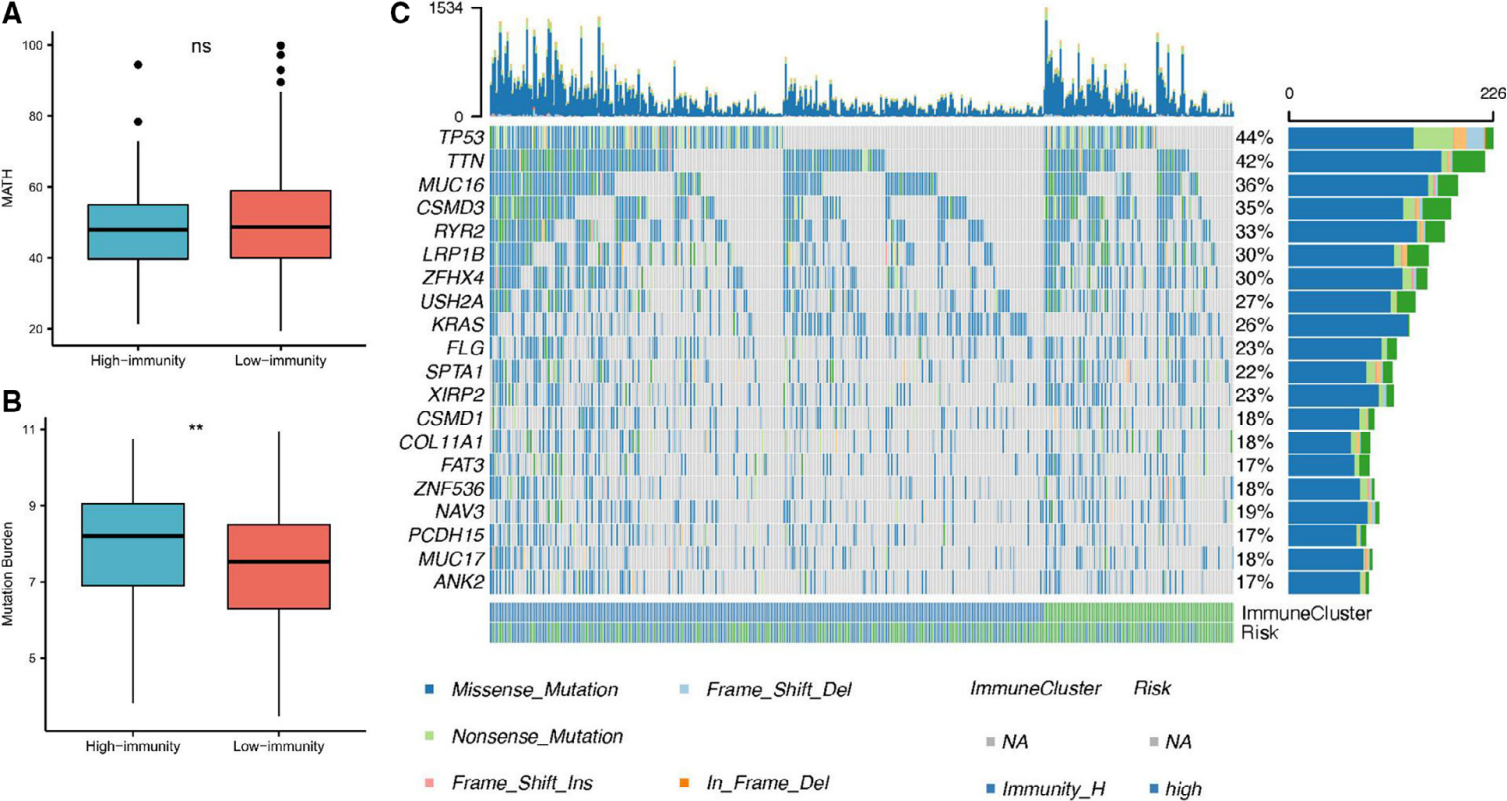
Somatic mutation of the immune cluster. (A) The heterogeneity of the mutant alleles in each patient was calculated by the "maftools" package in r version 3.4.2 (2020‐04). No statistically significant difference of the MATH was found in these two groups. (B) The tumor mutation burden was calculated based on the TCGA_MC3 maf file. The tumor mutation burden of the low‐immunity group was significantly lower than that in the high‐immunity group. (C) The landscape genome changes of LUAD were visualized by the Oncoprint r package. The Student’s *t*‐test was used to compare the difference between high‐ and low‐immunity groups (**P < 0.01). ns, not significant.

Because the tumor somatic mutation is an important factor for the treatment and survival of patients with cancer, we then calculated the tumor burden and tumor heterogeneity between the two immune clusters. Consequently, the tumor mutation burden of the low‐immunity group was significantly lower than that in the high‐immunity group, while no statistically significant difference of the mutant allele heterogeneity (MATH) was found in these two groups (Fig. 6A,B). In addition, the top 10 mutated genes are shown in Fig. 6C.

### GSEA for different immune clusters of LUAD

**Fig. 7:**
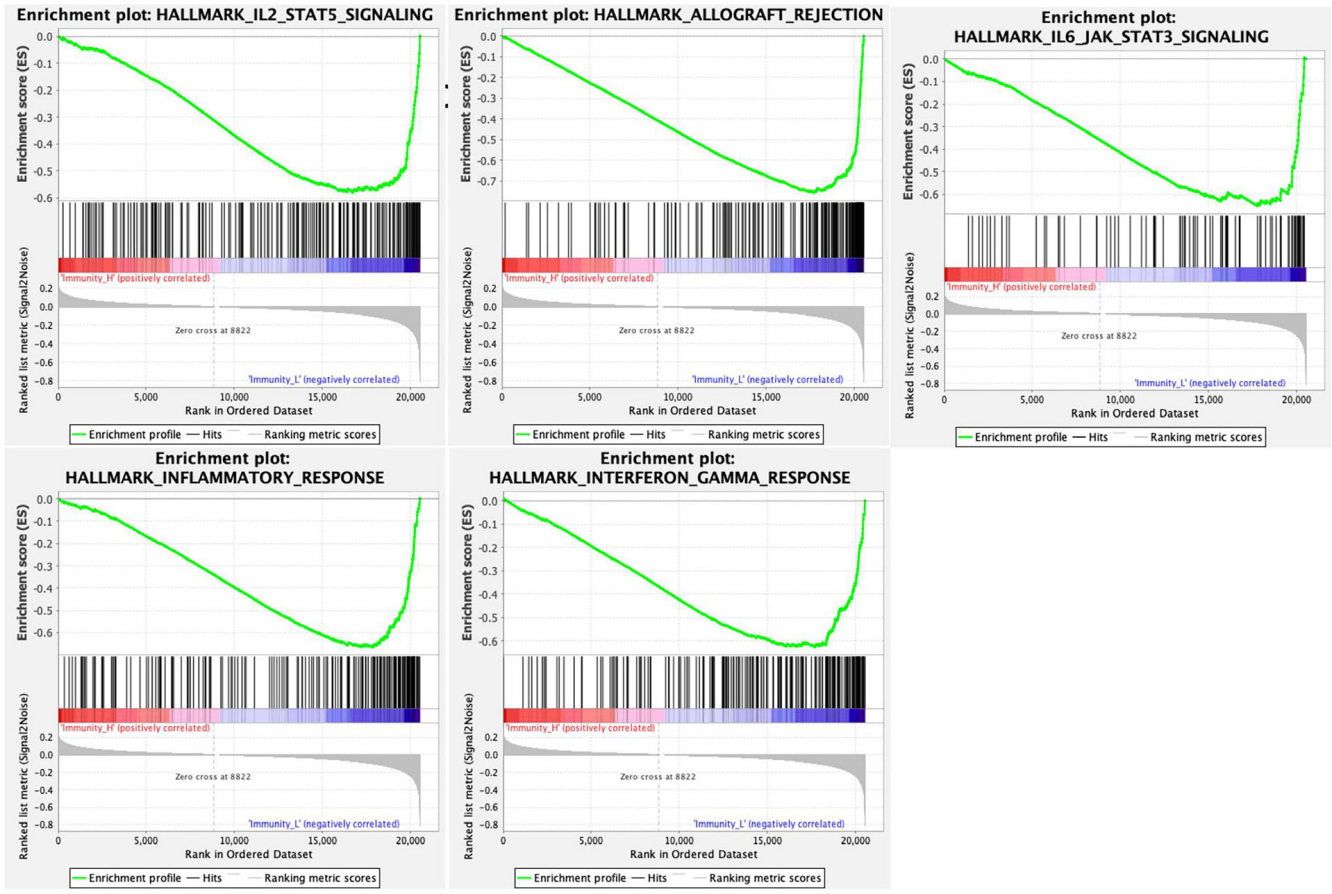
GSEA for immune clusters of LUAD. The GSEA was performed for the immune clusters. The number of permutations was set at 1000. The significance level of the gene sets was set at |NES| > 1, NOM *P* < 0.05 and FDR *q* < 0.25. Notably, the several immune‐related gene sets were related to the immune clusters, including IL‐2/STAT5 signaling, inflammatory response, IL‐6/JAK/STAT3 signaling, and interferon‐gamma response and allograft rejection.

According to the immune clusters, we divided the patients with LUAD into the low‐ and high‐immunity groups. As a result, none of the 50 gene sets was alternated in low‐immunity phenotype, and 42 gene sets were up‐regulated in high‐immunity phenotype. Of the results, 17 gene sets were significantly enriched at FDR < 25%, and 13 gene sets were significantly enriched at a nominal *P* < 5% (Table 2). Notably, the several immune‐related gene set enrichments were related to the immune clusters, including interleukin‐2 (IL‐2)/STAT5 signaling, inflammatory response, IL‐6/Janus kinase(JAK)/signal transducer and activator of transcription 3(STAT3) signaling, interferon‐gamma response and allograft rejection (Fig. 7). In addition, the alternated cancer‐related hallmark pathway was also found, such as KRAS signaling up, epithelial–mesenchymal transition and phosphatidylinositol‐3‐kinase (PI3K)/Akt and the mammalian target of rapamycin (mTOR) signaling. These results might provide evidence for the treatment of patients with LUAD with different immune clusters.

**Table 2:** Significant gene set enrichment in the high‐immunity group. ES, enrichment score; FWER, family‐wise error rate; GS, gene set.

GS details	Size	ES	NES	NOM *P*‐value	FDR *q*‐value	FWER *P*‐value	Rank at max
HALLMARK_COMPLEMENT	195	−0.59	−1.96	0.000	0.056	0.053	4669
HALLMARK_ALLOGRAFT_REJECTION	195	−0.76	−1.90	0.000	0.041	0.081	2811
HALLMARK_PI3K_AKT_MTOR_SIGNALING	103	−0.39	−1.96	0.002	0.110	0.052	930
HALLMARK_IL2_STAT5_SIGNALING	194	−0.58	−1.94	0.002	0.045	0.063	3828
HALLMARK_INFLAMMATORY_RESPONSE	197	−0.66	−1.75	0.002	0.091	0.206	2669
HALLMARK_IL6_JAK_STAT3_SIGNALING	87	−0.65	−1.78	0.004	0.093	0.181	2767
HALLMARK_KRAS_SIGNALING_UP	193	−0.56	−1.70	0.004	0.091	0.278	4136
HALLMARK_EPITHELIAL_MESENCHYMAL_TRANSITION	194	−0.66	−1.74	0.014	0.086	0.219	4669
HALLMARK_APOPTOSIS	158	−0.43	−1.68	0.018	0.091	0.303	3717
HALLMARK_UV_RESPONSE_UP	152	−0.36	−1.55	0.020	0.192	0.510	5528
HALLMARK_INTERFERON_GAMMA_RESPONSE	196	−0.63	−1.70	0.028	0.100	0.272	2841
HALLMARK_APICAL_SURFACE	43	−0.53	−1.51	0.030	0.178	0.577	3815
HALLMARK_HYPOXIA	190	−0.41	−1.54	0.034	0.187	0.524	4574

### Clinical impact of immune clusters on patients with LUAD

**Fig. 8:**
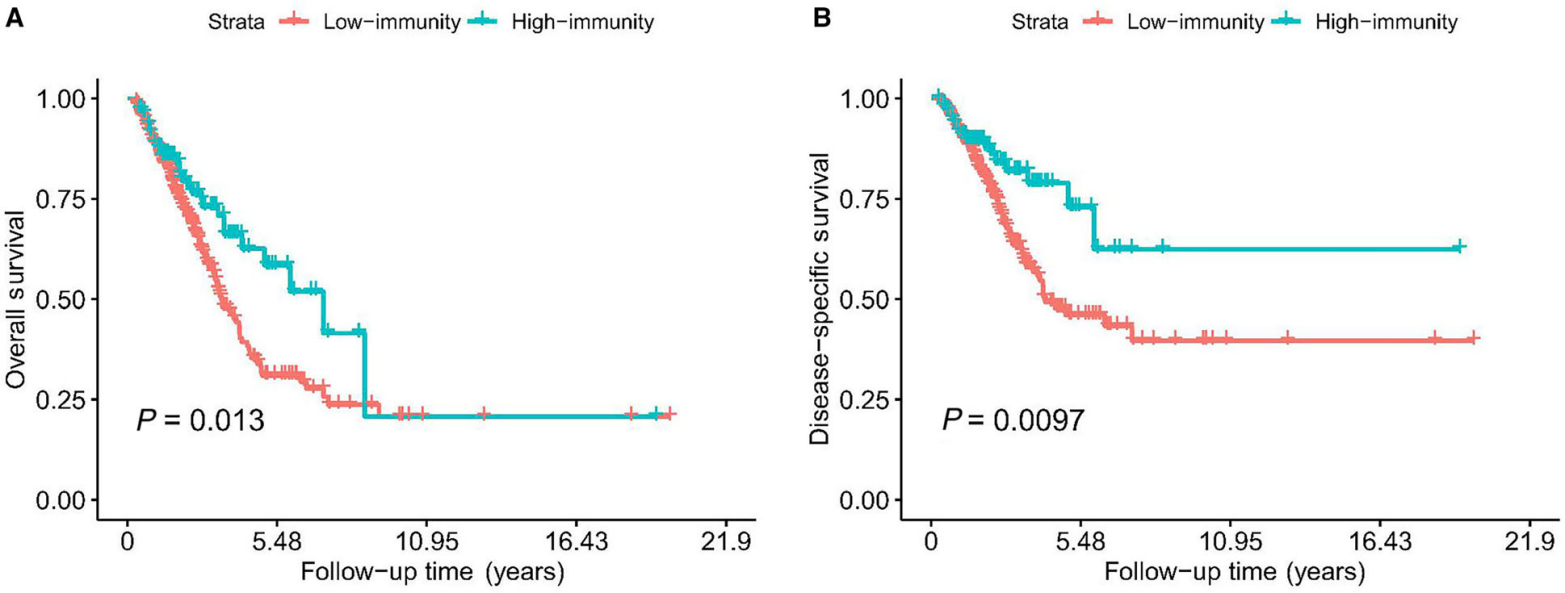
Prognostic value of immune subtypes for patients with LUAD. (A) Overall survival. The overall survival period of high‐immunity patients was higher than that of the low‐immunity patients (log rank *P* = 0.013). (B) Disease‐free survival. The disease‐specific survival outcome of high‐immunity patients was more favorable than in patients with low‐immunity (log rank *P* = 0.0097).

Finally, we also explored the clinical implication of immune clusters in patients with LUAD by analyzing the phenotype data. As result, the frequency of patients with tumor stage I–II in the high‐immunity group was significantly higher than that in the low‐immunity group, suggesting that the tumor growth might be inhibited by high immunity (χ^2^ = 5.158, *P* = 0.023; Table S1). In addition, we also analyzed the prognostic value of the immune subtypes for patients with LUAD. As Fig. 8A shows, the overall survival period of the high‐immunity patients was higher than that of low‐immunity patients, with a log rank *P* value of 0.013. More importantly, the disease‐specific survival outcome of high‐immunity patients was more favorable than with low‐immunity patients (*P* = 0.0019; Fig. 8B). These results suggested that high‐immunity patients achieved a better survival outcome than the low‐immunity ones.

## Discussion

LUAD is the major subtype of lung cancer and one of the leading causes of cancer deaths worldwide [20]. Due to the potential toxicity and side effects of some drugs, patients often fail to obtain consistent therapeutic efficacy from drugs. Furthermore, the application of postoperative systemic adjuvant chemotherapy is still controversial in clinical practice [21]. Therefore, better prognostic tools for LUAD are necessary.

This study established a regulatory network of TFs and a prognostic model for LUAD while combining clinical factors to verify potential prognostic markers. TFs recognize DNA sequences that control gene expression and are important regulators of cellular function and responses to environmental stimuli [22]. We found positive regulatory relationships between the following TFs and immune genes: *ETV1* and *SCH3*, as well as *IRF4* and *IGKV4‐1*. TFs that were positively regulated by the immune gene *VIPR1* included *EPAS1*, *TCF21* and *CBX7*. Studies have shown that the TF ETV1 [23] increases the risk for carcinogenesis by increasing NSCLC proliferation; furthermore, it is negatively correlated with overall survival in patients with NSCLC. The expression of ETV1 is higher within tumor cells compared with normal tissues. In the study by Qian *et al*. [24], IRF4 was confirmed to be an important regulator of cell growth in NSCLC. In the study by Chen *et al*. [25], IRF4 was associated with unfavorable outcomes among patients with NSCLC. These findings may indicate that IRF4 reflects the activity of tumor‐infiltrating lymphocytes in tissue sections. Studies have shown that EPAS1 is associated with treatment resistance, metastasis and poor clinical prognosis among patients with lung cancer [26, 27]. Furthermore, high levels of EPAS1 protein were associated with poor prognosis in NSCLC [28, 29]. The low expression of TCF21 is an important factor for poor prognosis of LUAD, but not lung squamous cell carcinoma [30]. Also, CBX7 is significantly down‐regulated in lung cancer [31]. Therefore, we can use these TFs related to LUAD prognosis for new therapeutic targets.

The combined analysis of prognostic biomarkers and TME may provide insight into novel molecular mechanisms and ways to improve the management of immunotherapy patients [31, 32]. In this study, 29 sets of immune genes containing immune cell types and immune‐related functions and pathways were downloaded from the literature. These data were used to study the relationship between 15 genes and TME among patients with high versus low immune scores or stromal score groups using the ESTIMATE algorithm. Previous studies have reported that 9 out of the 15 immune genes associated with prognosis (*S100A16*, *CRABP1*, *RBP2*, *FGF2*, *FPR2*, *BDNF*, *ANGPTL4*, *SEMA4B* and *VIPR1* [33, 34, 35, 36, 37, 38, 39, 40, 41]) are involved in the pathogenesis of lung cancer. The remaining six genes (*IGKV4‐1*, *IGKV6D‐41*, *TNFRSF11A*, *INHA*, *IL11* and *SCH3*) have not been reported in the literature and may be used as potential biomarkers for lung cancer research. Moreover, immune gene prognostic models for LUAD predict patient survival in combination with clinical characteristics and TME. Because only retrospective LUAD data from TCGA database were analyzed, further validation of clinical samples is needed. Although Seo *et al*. [14] have conducted the article focused on the subtypes of NSCLC, there were several differences between this previous study and our work. First, the previous study constructed the subtype based on 87 LUAD samples, which were comparatively few. Our study investigated the immune subtype based on a total of 447 LUAD samples, of which the results might be more stable. Second, the previous study clustered the patients into different subtypes based on the 1000 most variable genes identified by Principal Component Analysis (PCA). We considered whether these 1000 most variable genes could present the most variable characteristics for the immune state, or whether they might contribute more representative characteristics to the other subtype. As we acknowledge, theintratumor heterogeneity and mutant allele tumor heterogeneity are the important factors for the TME [42, 43]; thus, we calculated their association with the immune subtypes. More importantly, the tumor mutation burden has proved to be a useful predictive biomarker for immune therapy, which is strongly relevant to the immune status [44]. What is more, our work also investigated the enrichment of different subtypes by GSEA instead of the methods used in the previous study based only on the differentially expressed genes [45]. Altogether, our work is radically different from the previous study, which described the immune subtypes of LUAD more comprehensively and systematically.

## Conclusions

In summary, this immune gene prognosis model identifies new targets for the treatment of LUAD. We hope that these immune subtypes will be a guide for determining the survival, prognosis, clinical diagnosis and treatment of different LUAD immunophenotypes.

## Author contributions

JW and LY performed data analyses and helped prepare the manuscript. YS provided study materials. FQ, ZX and WC conceived the research, determined the appropriate analyses to be performed and wrote the manuscript. SL designed this study. All of the authors read and approved the final manuscript.

## Conflict of interest

The authors declare no conflict of interest.

## Supporting information


**Table S1.** Associations of the immune subtypes and the clinical features of LUAD.

## Data Availability

The data were freely available in TCGA (https://portal.gdc.cancer.gov/). The data are available from the corresponding author upon reasonable request.
